# Management of Juvenile Myasthenia Gravis

**DOI:** 10.3389/fneur.2020.00743

**Published:** 2020-07-24

**Authors:** Karen O'Connell, Sithara Ramdas, Jacqueline Palace

**Affiliations:** ^1^Nuffield Department of Clinical Neurosciences, John Radcliffe Hospital, University of Oxford, Oxford, United Kingdom; ^2^Department of Paediatric Neurology, John Radcliffe Hospital, Oxford, United Kingdom

**Keywords:** juvenile myasthenia gravis, treatment, thymectomy, immunosuppression, autoantibodies, generalized myasthenia gravis, ocular myasthenia gravis, myasthenic crises

## Abstract

Juvenile Myasthenia Gravis (JMG) is a rare disorder, defined as myasthenia gravis in children younger than 18 years of age. While clinical phenotypes are similar to adults, there are a number of caveats that influence management: broader differential diagnoses; higher rates of spontaneous remission; and the need to initiate appropriate treatment early, to avoid the long-term physical and psychosocial morbidity. Current practice is taken from treatment guidelines for adult MG or individual experience, with considerable variability seen across centers. We discuss our approach to treating JMG, in a large specialist JMG service, and review currently available evidence and highlight potential areas for future research. First-line treatment of generalized JMG is symptomatic management with pyridostigmine, but early use of immunosuppression, where good control is not achieved is important. Oral prednisolone is used as first-line immunosuppression with appropriate prevention and monitoring of side effects. Second-line therapies including azathioprine and mycophenolate may be considered where there is: no response to steroids, inability to wean to a reasonable minimum effective dose or if side-effects are intolerable. Management of ocular JMG is similar, but requires close involvement of ophthalmology in young children to prevent amblyopia. Muscle-specific tyrosine kinase (MuSK)-JMG show a poorer response to pyridostigmine and anecdotal evidence suggests that rituximab should be considered as second-line immunosuppression. Thymectomy is indicated in any patient with a thymoma, and consideration should be given in acetylcholine receptor (AChR) positive JMG allowing time for spontaneous remission. The benefit is less clear in ocular JMG and is not advised in MuSK-JMG. Children experiencing a myasthenic crisis require urgent hospital admission with access to the intensive care unit. PLEX is preferred over IVIG due to rapid onset of action, but this needs to be balanced with feasibility in very young children. Key questions remain in the management of JMG: when to initiate both first- and second-line treatments, choosing between steroid-sparing agents, and determining the optimal dose and treatment duration. We feel that given the rarity of this disease, the establishment of national registries and collaboration across groups will be needed to address these issues and facilitate future drug trials in JMG.

## Introduction

Autoimmune myasthenia gravis (MG), is a disorder of neuromuscular transmission, resulting from antibodies to components of the muscle endplate that cause impaired synaptic transmission. In the majority of cases, these antibodies are directed toward the acetylcholine receptor (AChR), but they can also target muscle specific kinase (MuSK) and possibly to receptor related low density lipoprotein-4. The clinical hallmark is fatigueable muscle weakness which can be limited to the ocular muscles or more generalized. Juvenile Myasthenia Gravis (JMG) is defined as myasthenia gravis in children younger than 18 years of age. While clinical phenotypes are similar to adults, there are a number of caveats unique to JMG, that need to be considered when evaluating these patients. In this article, we will give a brief overview of the epidemiology, pathophysiology, clinical presentation, and diagnosis of JMG and a more comprehensive review of currently available therapies and approach to management, and finally outline our JMG treatment paradigm.

## Epidemiology of JMG

Population-based studies to determine the incidence of JMG demonstrate the rarity of this disorder, and racial variability. A large nationwide study in the UK identified 101 children (<18 years) who had antibody positive JMG (with 95% AChR and 5% MuSK antibodies), equating to an incidence rate of 1.5/million person years (1). Of this cohort only 20% were under 10 years of age when diagnosed. Similarly, a national Norwegian study of 43 incident cases, showed an incidence rate of 1.6/million person years but this cohort included both seropositive and seronegative cases (2). Again lower rates were seen in pre-pubertal children (<12 years) of 0.9/million person-years. In a long-term follow-up study in Denmark from 1996-2009, the incidence rate was 7 times higher in those aged 10–19 years compared to those aged 0–9 years (2.2/million v 0.3/million person-years) (3). The incidence in Olmstead county, USA, was determined as part of a larger cohort study as 1.2/million person years but this was only based on two confirmed cases (4). In contrast to these studies, a national study of AChR antibody positive MG from South Africa, estimated to account for 75% of cases, showed a higher incidence rate of 4.3/million person-years in those under 20 years (5). Similar rates were seen in those under 10 years and those aged 10–14 years, of 3.3 and 2.9/million person-years, respectively. A large population-based study in Southern China showed that 45% of cases had onset in childhood (<14 years) (6). In Taiwan, incidence was highest amongst the 0–4 year age group at 8.9/million cases, dropping to 3.7/million in the 10–14 year age group (7). This peak in the 0–4 year age group, was also seen in a nationwide Japanese survey of MG prevalence (8). Taken together these studies highlight racial differences in JMG, with higher rates overall, and in children under 10 years, seen in the predominantly Black South African population, and amongst Chinese, Taiwanese and Japanese populations, when compared to European studies of majority Caucasian populations (1–3, 5, 7).

In a UK cohort study of JMG, the proportion of Black and Asian children was disproportionately raised compared to the background population of the UK, supporting these differences are driven at least in part by genetics, rather than geographical location (9). Higher rates in females were seen in all studies, except in the pre-pubertal South African population, again indicating how race influences JMG epidemiology.

## Pathophysiology of JMG

MG is mediated by antibodies to components of the neuromuscular junction which disrupt synaptic transmission. In the majority of cases of MG, pathogenic antibodies to the nicotinic AChR are seen, which induce loss of functional AChRs, through a number of mechanisms. The principle effect is through complement-mediated destruction of the motor end-plate (10). They also cause internalization, and subsequent degradation of the AChRs and can directly interfere with ACh-binding to the receptor (11, 12). In MuSK MG, the exact pathogenic mechanism is less clear but felt to be different to AChR-Ab (12, 13). MuSK antibodies are monovalent and largely of the non-complement fixing IgG4 sub-class, so are unable to bind complement or cross-link and internalize AChRs. Passive and active transfer experiments have shown a reduction in AChRs (14). It has been proposed that MuSK-Ab, act pre-synaptically, interfering with LRP4 function, with the consequent dispersal of AChR clusters (15).

## Clinical Features of JMG

JMG can exist as a purely ocular form, or with more generalized skeletal muscle involvement. In children, the majority will present with ptosis and a variable degree of ophthalmoplegia, which can be markedly asymmetric which may help distinguish from genetic causes of myasthenia (16, 17). The ocular features can be mild and variable and it is important to assess for fatigue with prolonged up gaze (1 min) during the clinical examination. There are a number of useful signs on examination, that can be supportive of ocular MG, with variable sensitivity, including: Cogan's lid twitch sign (a brief twitch of the eyelid as it overshoots, as eyes return to the primary position from prolonged downgaze), improvement in ptosis after orbital cooling (placing an icepack over the eye for up to 5 min), and the “curtain sign” (worsening ptosis in the least affected eye, after lifting the worst affected eye) (18). Recognition, appropriate treatment, and prompt referral to ophthalmology is important to avoid long-term sequelae such as strabismus and amblyopia (19). In more generalized forms, patients may note proximal muscle weakness manifesting as difficulty getting up from floor, running, going up stairs, or lifting their arms above their heads. They may also have signs of bulbar and respiratory involvement including dysarthria, taking long periods to complete meals, difficulty swallowing, and shortness of breath. Symptoms may fluctuate throughout the day, but are typically better in the morning or after periods of rest. In MuSK-JMG, onset is said to be typically acute with predominant bulbar involvement and early respiratory crises, although ocular onset which often then spreads to become more generalized is not uncommon (13). The presence of both myokymia and fasciculations can also be indicative of this condition. Similar to adult MuSK-MG there is a strong female predominance with 89% of cases female in the largest published pediatric case series (20).

Children presenting with purely ocular JMG, may go on to develop generalized JMG. The majority of cases who convert do so within 6 months of symptom onset and occurs rarely if ocular symptoms persist in isolation for longer than 2 years (17, 21). The prevalence of pure ocular JMG and the proportion who convert to generalized JMG varies across studies and appears to be strongly influenced by race, with pure ocular JMG accounting for up to 90% of Asian cohorts (8, 9, 21–23). Ocular JMG is also associated with younger age at onset with higher rates seen in pre-pubertal children, regardless of race (9, 21–24). Post-pubertal JMG more closely mirrors adult MG with a greater proportion of generalized onset and lower rates of spontaneous remission.

Remission rates vary across studies but are generally higher than in adult populations. In a large Norwegian case series 5 out of 63 experienced a spontaneous remission, accounting for 14% and 5% of the pre- and post-pubertal cohorts, respectively (21). In the same cohort 51% achieved complete stable remission (CSR) defined as off treatment for at least 1 year and no signs or symptoms of MG, again higher in pre-pubertal children. In an English series of 74 patients, 23% achieved CSR (9). Antibodies to clustered AChR proved to be the only significant predictive factor of a drug-free remission. Only 17%, of 424 Chinese children, who were followed up for a minimum of 5 years, achieved a CSR (22). Interestingly, 55% had achieved CSR for a minimum of 12 months during the course of their follow-up, but this was not sustained, highlighting the relapsing nature of MG. Discontinuing medication was reported as the commonest triggering event, although no information was available on the speed at which the treatments were withdrawn and the time from discontinuation to symptom onset varied hugely, at 1 month to 21 years.

A broad differential exists in children including congenital myopathies, mitochondrial cytopathies, acquired demyelinating neuropathies, and congenital myasthenic syndromes and careful evaluation particularly in pure ocular forms or antibody negative cases is needed. Features that may suggest an alternative diagnosis include positive family history, presence of symptoms from birth or early infancy, muscle contractures, scoliosis, and no response to symptomatic or immunosuppressive therapies.

Transient neonatal myasthenia results from the passive transfer of maternal antibodies *in utero* and has been reported in 10–20% of children born to mothers with MG (25). Infants can present with generalized hypotonia, weak cry, poor suck, ptosis, and in rare cases respiratory insufficiency that may require ventilation. It is usually self-limiting, with symptoms typically beginning 48 h after birth and in general resolve over weeks to months. In rare cases a persistent myopathy has been described (26). It is felt to be due to the loss or inactivation of AChR at a critical time during fetal development and has been termed fetal AChR inactivation syndrome (FARIS).

## Diagnosis

The diagnosis of JMG is primarily based on the clinical picture, but positive antibodies and abnormal neurophysiology can support the clinical impression.

### Serology

Serological testing is useful adjunct in the diagnosis of JMG. Autoantibodies targeting the AChR are the most common and there are a number commercially available tests using radioimmunoprecipitation assay (RAI) or enzyme linked immunosorbent assay techniques. In JMG cohorts the frequency of AChR antibodies can vary from 70 to 80%, and is typically lower than adult MG cohorts (9, 21, 24, 27). Antibodies are more likely to be seen in generalized JMG when compared to ocular JMG, and given the increased prevalence of pure ocular MG in JMG cohorts, this likely accounts for the higher rate of seronegative cases (9, 27). It is important to repeatedly test seronegative patients at 6 monthly intervals as delayed seroconversion can be seen up to 5 years after onset and particularly in pre-pubertal children (21, 28).

Cell-based assays which detect clustered AChRs are not commercially available but can increase the diagnostic yield in antibody negative cases (29). In a UK study of 74 JMG patients, 50% of seronegative cases were shown to have these antibodies on subsequent testing (9). A similar pattern was seen amongst a Chinese cohort, where 15/34 seronegative patients were positive for low-affinity AChR antibodies using a cell-based assay (30).

Patients who are negative for AChR antibody can also be tested for MuSK antibodies which account for 5–8% of all MG patients and presents with a distinct phenotype as previously discussed (13). Recently, a MuSK cell-based assay has been developed, which when combined with an IgG Fc gamma-specific secondary antibody, detected low-affinity MuSK antibodies in 14/169 seronegative patients (31). Sensitivity and specificity will need to be confirmed in further studies but represents a promising development in reducing the number of truly seronegative cases. Autoantibodies to low density lipoprotein 4 (LRP4), agrin, and ColQ have been described in association with MG, however, their specificity remains to be determined, pathogenic mechanisms have not been fully elucidated, and there has been no animal models showing disease in response to passive transfer of antibodies which is an essential criterion in determining whether antibodies are truly pathogenic (32).

### Neurophysiology

Neurophysiology can play an essential role in the diagnosis of neuromuscular dysfunction, but can be technically challenging in young children and results will depend on techniques available and operator skill (33). Both repetitive nerve stimulation (RNS) and single-fiber electromyography (SFEMG) are recognized screening tests for myasthenia. The sensitivity of SFEMG approaches 95% but requires volitional muscle activation, which is often not possible or inconsistent in young children. Stimulated potential analysis using concentric needle electrodes (SPACE), is an alternative technique where the nerve is stimulated and signals recorded, eliminating the need for patient co-operation and has been shown to be useful in children, with a sensitivity of up to 92% achieved in patients with JMG (34). Although more sensitive the authors find that the specificity of the SFEMG is lower than for RNS (the latter requiring >10% decrement with 3 Hz stimulation), such that a negative SFEMG is strong support against, and the presence of decrement on RNS strong support for, myasthenia. The sensitivity of RNS is increased if performed in a weak muscle when a negative test suggests the weakness may not be myasthenic and can be useful in the clinic when a MG patient has superimposed functional weakness.

### Edrophonium Testing

Edrophonium is a quick-acting, short-lasting, anticholinesterase inhibitor. It can be administered intravenously as a diagnostic test for JMG, where you would expect to see, transient improvement of symptoms. It is most useful, in the setting of ptosis, ophthalmoplegia or dysarthria, as these symptoms can be easily and quickly assessed. Its use in clinical practice is now limited, due to the potential side-effects including bradycardia, and increased reliability of neurophysiological tests and antibody testing. In order to carry out the test the child needs continuous cardiac monitoring with appropriate resuscitation equipment at the bedside.

## Treatment of JMG

There are no internationally accepted standards of care for JMG, although this issue was recently addressed and recommendations published by the European Neuromuscular Center workshop study group (35). Management should be delivered by a multidisciplinary team, encompassing pediatric neurology and ophthalmology services with expertise in JMG as well as physiotherapy, occupational therapy, speech and language therapy, dietetics, and psychology input. Treatment typically involves a combination of symptomatic and immunosuppressive therapies, with thymectomy in appropriate cases.

### Supportive Therapy

Despite limited published evidence, we feel supportive management should be initiated early in the disease course, both to manage the impact of the disease itself on physical and mental health but also to mitigate the potential medication side-effects, particularly of corticosteroids. It is important to consider early input from allied health, with regard to diet and lifestyle. Both children and adults need to be cautious to avoid excessive weight gain in the context of reduced physical activity and advised with regard to healthy snacks and increased fruit and vegetable intake. The benefits of physical activity need to be highlighted, usually in the form of a graded exercise program, being mindful to avoid excessive fatigable weakness. Close communication with schools is important, and educational care plans may need to implemented to ensure that these children do not become unduly disadvantaged in accessing the educational curriculum. Varicella vaccination should be considered prior to initiation of immunosuppression, if clinically safe to delay. Annual influenza vaccination with inactivated vaccine is recommended. A recent study, suggested an association between a live-attenuated Japanese encephalitis vaccine and the high prevalence rate of JMG in China (36). The hypothesis was supported by a mouse model but has not been replicated by other groups and at this time, we would not contradict the use of live-attenuated vaccines where required. Intercurrent illnesses should be managed promptly. Families need to be provided with a list of medications that affect the neuromuscular junction, with potential to worsen the condition, and should therefore be avoided (Table 1). Regular ophthalmology input is needed, given the risk of amblyopia in this population. Discussing the potential psychological impact in the early stages may lead to early recognition and management, preventing low mood, depression-related fatigue and tiredness being mistaken for myasthenic symptoms and subsequent overtreatment.

**Table 1:** List of medications that may cause worsening of underlying myasthenia gravis.

**Type of medication**	**Medication**
Antibiotics	Aminoglycosides—gentamicin, amikacin, streptomycin, telithromycin^*^ Quinolones—ciprofloxacin, levofloxacin Tetracyclines—tetracycline, doxycycline, minocycline
Antimalarials	Chloroquine, hydroxychloroquine
Anesthetic agents	Muscle relaxants—succinylcholine
Antihypertensives	Beta-blockers—propranolol, bisoprolol, sotalol, metoprolol Calcium-channel blockers—verapamil, lercandipine, amlodipine
Antiarrhythmics	Procainamide, Quinidine
Rheumatic drugs	Penicillamine
Immunotherapy	Checkpoint inhibitors
Antipsychotics	Chlorpromazine, risperidone
Miscellaneous	Magnesium salts, Botulinum toxin

*It is important to note that this list is not exhaustive and should serve as a guide. Not all medications will exacerbate myasthenic symptoms to the same degree, and some of these medications can be used cautiously in patients with MG without deleterious effects, particularly is their MG is well-controlled. Common examples in each class of medication are listed but any medication within a class should be considered as carrying the same risk*.

* *Has been associated with deaths in MG and should never be given*.

### Symptomatic Therapy

Cholinesterase inhibitors (ChE-I) are used first-line as symptomatic treatment in JMG. Pyridostigmine is a non-selective ChE-I and is the most widely used. These drugs act at the neuromuscular junction, where they interfere with the breakdown of acetylcholine (ACh) increasing its availability to bind to post-synaptic nicotinic receptors. A Cochrane review in 2014 concluded that the evidence from observational studies clearly show significant benefit in MG, and it would not be justified to conduct placebo-controlled study in this patient group (37). We advocate starting at 0.5–1 mg/kg, taken 3–4 times per day, and this can be increased to up to 1.5 mg/kg 5 times per day (maximum 450 mg/day). Times and doses can be adjusted to an individual child's time-table, such as taking their dose 30–60 min before significant physical activity. Slow-release forms of pyridostigmine are available but they come in high dose preparations (180 mg tablets) which limits their use in children and long acting preparations may build up over time and make it difficult to assess immediate response to medication. Failure to suppress symptoms with doses around 1 mg/kg 4 times per day, should lead to consideration of immunosuppressive therapy, taking into account the severity of symptoms. It should be noted that pyridostigmine works within hours and thus the drug effectiveness can be assessed within a couple of weeks. Decision to initiate immunosuppression, should not be delayed for several months, whilst increasing pyridostigmine to maximal tolerated dose. This can lead to a delay in resolution of weakness, and subsequently, impact on the social, and educational activities of the child.

Side-effects relate to excessive cholinergic stimulation, and include abdominal cramps, diarrhea, hypersalivation, sweating, blurred vision, bradycardia, hypotension, and bronchoconstriction. Anti-cholinergic medications that do not bind to the nicotinic receptor, such as propantheline and glycopyrrolate can be a useful adjunct to manage these symptoms and increase tolerability. It should be noted, that a poorer response to ChE-I has been seen in MuSK-MG patients with higher rates of side-effects on standard doses and in some cases clinical deterioration (13).

### Immunosuppressive Therapy

There are no formal guidelines for the use of immunosuppressive therapy in JMG and current practice has been taken from adult guidelines and expert opinions based on individual experience (35, 38, 39).

Despite the lack of clinical trials, prednisolone is accepted as the first-line immunosuppressive therapy in JMG (38). The recommended starting dose is 0.5 mg/kg alternate days. Higher doses can be associated with worsening of MG symptoms and should only be attempted in the in-patient hospital setting. Doses are gradually uptitrated, pending response, to a maximum of 1.5 mg/kg alternate days (maximum: 100 mg) or 1 mg/kg/day (maximum: 60 mg). Lower doses may be required in pure ocular JMG, although outcomes in patients with ophthalmoparesis were better, if patients were treated earlier with higher dose steroids (40). A benefit is usually seen within weeks but it can take up to 6 months or longer to see the full effect of a treatment dose. The goal of therapy is to induce remission and then to wean off ChE-I first and then slowly reduce the corticosteroids monthly to the lowest effective maintenance dose. We would typically reduce by 5 mg every month to 15–20 mg alternate days and then reduce by 1 mg per month to stop. If the maximum prednisolone dose has been used, then this wean can take over a year. Sequential or concurrent initiation of corticosteroids with ChE-I was discussed at a recent expert workshop in JMG, with the majority favoring a short trial of ChE-I prior to the introduction of steroids in mild JMG and a consensus that they should be commenced concurrently in moderate or severe JMG particularly if bulbar symptoms were present (35).

There a numerous adverse effects associated with steroid use, including mood and behavioral disturbance, sleep disruption, weight gain, growth restriction, hypertension, diabetes, osteoporosis, infections, and gastric-esophageal reflux disease (GORD). In order to mitigate these effects all children should be commenced on vitamin D as per local guidelines for bone health and consideration given to gastric protection. Children and their families need to be given advice on potential for weight gain, and healthy eating (increasing vegetable portions and healthy snacks) and exercise, discussed to prevent this. These side-effects can cause psychological stress, particularly in adolescents and psychological supports should be offered. Regular monitoring of blood pressure, growth velocity, and weight should be carried out while on treatment and those on long-term steroids should have a bone density assessment.

Second-line therapies or steroid-sparing agents may be introduced when (1) there is no response to steroids, (2) an inability to wean steroids to a reasonable minimum effective dose, or (3) if side-effects of steroid treatment become intolerable. These include but are not limited to azathioprine, mycophenolate mofetil, tacrolimus, rituximab, cyclosporine, and cyclophosphamide and use may vary depending on an individual center's experience. Intravenous immunoglobulin (IVIG) and plasma exchange (PLEX) have also been used as maintenance therapy (41).

Use of azathioprine in JMG is largely guided by expert opinion (35, 38, 39). A number of case series confirm its use, in both generalized and ocular JMG but none were designed to assess its efficacy (17, 27, 42, 43). It is a purine analog that acts by suppressing B cell and T cell proliferation. It is converted to its active metabolite 6-mercaptopurine by the enzyme thiopurine methyltransferase (TPMT). All patients should be screened for TPMT activity prior to starting azathioprine, as reports suggest enzyme deficiency, is more likely to be associated with myelosuppression. It is commenced at a dose of 1 mg/kg either daily or twice daily and can be increased by 0.5 mg/kg every 2–4 weeks to 2.5 mg/kg/day (35, 39). Azathioprine is typically used in combination with steroids and has been shown in a clinical trial of adult MG patients to be a useful steroid-sparing agent but its effects can take up to 12 months to become fully effective (44). The use of azathioprine may also allow a reduction in dose or tailing off of prednisolone.

The side-effects seen with azathioprine use include GI disturbance, liver dysfunction and myelosuppression. Patients should have a full blood count and liver function tests weekly, until on the maintenance dose for 8 weeks, and then 3 monthly if test parameters remain stable. Azathioprine is felt to be safe in pregnancy and thus is a good choice for female children of all ages, likely to need long term treatment (45).

Mycophenolate mofetil selectively inhibits B cell and T cell proliferation, by targeting cells that rely on the *de novo* pathway for purine synthesis. A large international phase III trial in adult MG patients failed to reach its primary endpoint, although the study period was likely too short at 36 weeks (46). This is supported by a retrospective study of 102 AChR-Ab positive patients, in which 80% of patients who were followed for longer than 24 months had improved, and 56% had been able to discontinue steroids (47). Some pediatric patients were included in this cohort but no breakdown of response was given. Given the lack of evidence, mycophenolate is considered a second-line agent and used if patients are intolerant or fail to respond to azathioprine. Common side-effects include nausea, vomiting, diarrhea, and less frequently leukopenia. Mycophenolate has been shown to be teratogenic and use is generally avoided in females of childbearing age as a long term option (48).

Tacrolimus is a calcineurin inhibitor which provides an immunosuppressive effect by modulation of T cell activity and support of antibody production in B cells. It is from the same class as cyclosporine but is felt to be less nephrotoxic. A systematic review of all prospective studies in adult MG suggested a beneficial effect on MG symptoms and facilitated the reduction of overall steroid dose (49). The studies were largely carried out in Asian populations which may limit the generalizability of these results. Long-term follow-up was available in some studies with no safety signal generated. An open-label trial in China, looked at the safety and efficacy of tacrolimus in 13 steroid-refractory JMG patients (50). The majority of patients had ocular MG, were aged 7–13 years and mean disease duration was 42 months. At 12 months follow-up, 10 patients were able to discontinue steroids and an improvement was seen on QMG and other quality of life measures. There is also a number of case reports that suggest a benefit in treatment refractory patients (51–54). Side-effects of tacrolimus include hypertension, headache, tremor, renal impairment, new-onset diabetes mellitus, diarrhea, malignancy (e.g., lymphoma and dermatologic), and increased risk of infection.

Rituximab is a chimeric monoclonal antibody, which acts by binding to CD20 on B cells and triggering cell death. It is given intravenously, at a dose of 375 mg/m^2^/week for 4 weeks or two doses of 750 mg/m^2^ (up to maximum 1 g) 2 weeks apart. If required, repeat doses may be given, typically when the CD19+ CD20+ B cells begin to rise (usually around 6 months). Its use in MG is typically reserved for treatment refractory cases. A recent systematic review of rituximab use in adult MG identified 108 adult MG patients, treated with rituximab in 9 case series and one uncontrolled trial (55). The review concluded that all studies demonstrated an improvement in MG symptoms and the majority of patients were able to reduce concomitant immunosuppressive drugs. MuSK-MG patients tended to respond better.

The pediatric literature for rituximab is limited to case series or case reports, and generally shows favorable results in treatment refractory JMG (28, 42, 56–58). The largest series, reported on rituximab use in 5 children with refractory JMG, 3 AChR-Ab positive, and 2 MuSK-Ab positive (42). It was described as well-tolerated and two children improved significantly, while the remainder had a partial response. Complete remission has been reported in one case of MuSK-JMG (59). Side-effects were not reported but rituximab has been associated with higher rates of infection including opportunistic infections such as progressive multifocal leukoencephalopathy (PML), and may cause long-term B cell depletion and hypogammaglobulinaemia. These studies, taken together, support a role for rituximab in the treatment of refractory JMG, particularly MuSK-JMG, but there a number of issues around its use that are unresolved including: duration of treatment, timing of future doses, and repeating cycles in cases where there is no clear response.

Cyclosporine, methotrexate and cyclophosphamide can be used as alternative immunosuppressive agents in MG but typically in treatment refractory cases, where other options have failed, due to lack of confirmed efficacy or concern around the side-effect profile. Cyclosporine has been shown to be effective as a steroid-sparing agent in adult MG patients but high rates of side-effects, particularly renal toxicity limit its use (60, 61). Efficacy of methotrexate as a steroid-sparing agent in adult MG was shown in an uncontrolled trial, but a more recent randomized controlled study failed to achieve this primary end-point, although the authors argue the study methodology may have been flawed (62, 63). Cyclophosphamide has been shown to be effective in inducing remission in treatment refractory adult MG patients, but is associated with high relapse rates unless used in conjunction with other immunomodulating therapy (64–66). There are significant side-effects associated with cyclophosphamide use, including bladder cancer and hematological malignancies, as well as possible implications on fertility. The risk is determined by the cumulative dose over time, and all patients need to be counseled with regard to these risks prior to initiating therapy. There are no studies looking at outcomes of these medications in JMG.

Intravenous immunoglobulin (IVIG) is used in many neurological conditions due to its diverse mechanisms of action (67). It has been shown to inhibit complement binding, neutralize pathogenic cytokines, downregulate antibody production, enhance remyelination and modulate Fc-receptor-mediated phagocytosis, and T cell function. Response to treatment is typically seen within days but can take a couple of weeks to be maximal. These characteristics mean it is useful in treating exacerbations or to optimize function prior to thymectomy as demonstrated in case series of JMG patients (41, 68, 69). It has also been shown to be effective as a maintenance therapy but availability due to a worldwide shortage and resource implications due to high cost need to be considered (41). The typical dose is 1 g/kg given intravenously and repeated over 2 days. In general the maintenance dose is 1 g/kg repeated at 4–6 weekly intervals but this will depend on patient response. Side-effects include infusion reactions, rash, headache, hypertension, increased risk of thrombosis and aseptic meningitis.

The principal mechanism of action of plasma exchange (PLEX) is the removal of pathogenic autoantibodies from the circulating blood stream (70). It has also been suggested that it may affect lymphocyte proliferation and function. The indications for use are similar to IVIG, mainly in the treatment of exacerbations and inducing stability pre-surgery. Although studies in adult patients, have shown no difference in clinical outcomes with either IVIG or PLEX (71) most myasthenia experts feel PLEX is probably more effective in most patients and may be quicker acting in practice. In a study comparing outcomes between generalized JMG patients treated with either PLEX or IVIG as a maintenance therapy, a higher response rate to treatment was seen in PLEX group but numbers in each group were small (17 in total) (41). Favorable outcomes have also been seen in MuSK-MG. In a multicentre study of 110 patients, improvement was seen in 93% of those who were treated with PLEX compared to 61% who received IVIG (72). The response to PLEX was described as rapid and thus is preferred in the treatment of exacerbations. A typical course of treatment is 3–5 exchanges on alternate days and often requires placement of a central venous catheter. The major limiting factor in small children is inadequate venous access and as such IVIG may be more practical in that setting.

### Thymectomy

The role of the thymus in MG pathogenesis is supported by a number of factors, including the high rates of thymic pathology seen in MG patients, correlation between anti-AChR antibody levels and the degree of follicular hyperplasia and favorable outcomes post-thymectomy (73). Thymic hyperplasia is not uncommon in JMG but thymoma is rare as demonstrated by a number of large cohort studies (22, 74, 75). All children should have thymic imaging (CT or MRI) regardless of clinical presentation.

The presence of a thymoma is an absolute indication for thymectomy, but its role in non-thymomatous MG depends on the antibody status, the age and disease duration, and subtype of MG. A benefit has been shown in a single international randomized controlled trial of transsternal thymectomy of adult MG patients (aged 18–65 years), which demonstrated better clinical outcomes and reduced medication requirements, in those who underwent thymectomy and corticosteroids compared to corticosteroids alone (76). All patients within this study had AChR-Ab positive generalized MG and were within 5 years of diagnosis. Now that VATs thymectomy is the surgical technique of choice, with lower associated morbidity, it may be considered in patients outside those included in the trial i.e., those with ocular MG, and those without detectable AChR-Abs who are MUSK antibody negative (such patients may have undetectable AChR-Abs and thymic hyperplasia) (77). While there has been no trials in pediatric patients, a recent systematic review, which included 488 patients who underwent thymectomy, showed that the procedure was well-tolerated and 77% symptomatically improved after the surgery (78). Furthermore, sustained remission was seen in 29%. Patients with pure ocular symptoms accounted for half of the total cohort. A small number of studies, have attempted to compare surgical and non-surgical management, with discordant results, but these retrospective cohorts were not matched for age, sex, disease features, duration of symptoms, etc. limiting the generalizability of results (21, 78). A recent study showed that thymectomy did not influence conversion from ocular to generalized disease (22).

Despite the lack of prospective studies evaluating thymectomy in JMG, it is generally accepted that thymectomy is considered as part of the initial management of all AChR-Ab positive generalized JMG patients. Its role is less clear in children with milder disease due to the higher rate of spontaneous remission in this group. In our opinion, it should also be considered in AChR-Ab positive ocular JMG patients, who fail to respond to a reasonable trial of immunosuppression, to avoid the long-term sequelae of these treatments. Earlier surgical intervention (within 2 years of symptom onset) has been associated with better outcomes (79). This needs to be balanced against patient age; with higher rates of spontaneous remission seen in pre-pubertal children and also allowing time for immune maturation in very young children. A recent review, of neonatal thymectomy for congenital heart disease, has shown that in the short-term, the rate of infections and autoimmunity do not appear to be increased in this patient cohort but long-term follow-up studies are lacking (80). Patients with MG are at an increased risk of developing other autoimmune diseases, in particular autoimmune rheumatological disease (ARD) including rheumatoid arthritis (RA) and systemic lupus erythematous (SLE) (81). In a large Taiwanese study looking at 6478 MG patients, the risk of developing an ARD was 6 times higher than age- and sex-matched controls. Analysis of those who underwent thymectomy demonstrated risk was 10 times higher than age- and sex-matched controls and no increased risk was seen in those who underwent PLEX. It needs to borne in mind, that those undergoing thymectomy are more likely to be antibody positive and have thymic hyperplasia, which may be independent risk factors for developing other autoimmune diseases. There is no indication for thymectomy in MuSK-JMG and its role in seronegative cases is unclear.

Further advances have seen a move toward minimally invasive techniques which demonstrate similar clinical outcomes and have the advantage of lower morbidity and shorter length of stay (82, 83). The limitation of these techniques is that it may not always be possible to achieve a complete resection.

### Other Potential Treatments

3,4-diaminopyridine (3,4-DAP) is a non-specific voltage-dependent potassium channel (Kv1.5) blocker, which causes depolarization of the presynaptic membrane at the NMJ and delays nerve repolarization, thus increasing quantal release of ACh. It is used in the treatment of Lambert-Eaton myasthenic syndrome (LEMS) and congenital myasthenia (84, 85). A recent phase IIb study in 10 adult patients with MuSK-MG, showed that it was safe and an improvement was seen across both objective measures of muscle strength and patient reported outcomes (86). The only treatment-related side-effect was transient paresthesia, which were reported in 60%, but did not lead to discontinuation of treatment.

Eculizumab is a humanized monoclonal antibody that targets complement protein C5 and inhibits terminal complement-mediated damage at the neuromuscular junction. It is licensed by both the Food and Drug Administration (FDA) in the United States and the European Medicines Agency (EMA), for use in adults with refractory AChR-Ab positive generalized MG, following a large multi-center RCT (87). Despite the study not achieving its primary end-point, benefit was suggested in the secondary outcomes including QMG scores and quality of life measures. The high cost will likely limit its use. A clinical trial in pediatric patients is currently underway (NCT03759366). As MuSK antibodies are predominantly IgG4, which do not activate complement pathways, Eculizumab may not be an effective treatment in this group.

### Management of Myasthenic Crises

Myasthenic crises result from significant neuromuscular weakness causing respiratory failure and a need for respiratory support (35, 38, 39). The frequency of myasthenic crises in JMG is unknown but accounted for 10% in one case series (21). Features which are suggestive of an impending crisis include worsening bulbar dysfunction, drowsiness, dyspnoea, and marked global weakness. Children who display any signs of impending crisis need to be urgently assessed in a unit where ventilatory support is available if needed. In the early phase of respiratory failure non-invasive ventilation may be an option, but with established infection and atelectasis endotracheal intubation is likely to be needed (88). The first step is to identify any potential triggers. They need to be screened for underlying infections including aspiration pneumonia. Any recent changes in medications must be noted. A number of commonly used medications including some antibiotics can exacerbate myasthenic symptoms. A list has been outlined in Table 1. Recent dose adjustments and compliance with myasthenia therapy should also be assessed as a rapid up-titration or withdrawal can also trigger a flare-up of symptoms. Both PLEX and IVIG can be used acutely and the regime has been discussed in the above paragraphs. PLEX may be favored due rapid onset of action but venous access can be an issue in small children. Cholinesterase inhibitors can be held while ventilated, and steroids can be started at the top dose without concern of deterioration because they will have respiratory support.

### Prescribing Considerations in Females of Child-Bearing Age

Given the high proportion of JMG in adolescent females, this is an important consideration when prescribing long-term immunosuppression in women of childbearing age. Clinical guidelines have been endorsed and published by the Association of British Neurologists (89). Pyridostigmine does not cross the placenta and has not been associated with fetal malformations and can be continued during pregnancy. The use of prednisolone, azathioprine and ciclosporin is also felt to be safe during pregnancy but mycophenolate and methotrexate should be avoided. The evidence on tacrolimus is less clear and the current license advocates use in pregnancy only, when no safer alternative is available. Data from transplant registries suggest no increased risk of congenital malformations, but high rates of pre-term delivery and low birthweight were seen, although both are common in this population who are often treated with multiple immunosuppressants (90). There are also a number of reports of transient hyperkalaemia in the neonate.

### Summary of Treatment Recommendations

- JMG needs to be managed by a multidisciplinary team.
- Supportive management should be instituted early to improve both physical and psychological outcomes.
- First-line symptomatic treatment is with ChE-I, most commonly pyridostigmine starting at a dose of 0.5–1 mg/kg, taken 3- 4 times per day, and this can be increased to up to 1.5 mg/kg 5 times per day (maximum 450 mg/day).
- Corticosteroids are used as first-line immunosuppressive therapy. They should be gradually increased to a maximum of 1.5 mg/kg alternate days (maximum: 100 mg) or 1 mg/kg/day (maximum: 60 mg). In any child with significant weakness or bulbar symptoms, admission to hospital for rapid escalation of steroid treatment may need to be considered.
- Second-line therapies including azathioprine and mycophenolate may be considered where there is: no response to steroids, an inability to wean to a reasonable minimum effective dose or if side-effects are intolerable.
- There is evidence to suggest rituximab may be more effective in MuSK-MG and may be considered as second-line therapy.
- While IVIG and PLEX can be used as maintenance therapies, they are generally reserved for treatment of acute exacerbations or to optimize function prior to surgery due to accessibility and resource constraints.
- Thymoma is an absolute indication for thymectomy and consideration should be given to all patients with generalized JMG who are AChR positive. The role in ocular and seronegative cases is less clear and there is no indication in MuSK-JMG.
- Children experiencing a myasthenic crisis or with significant weakness require urgent hospital admission with access to the intensive care unit. PLEX is preferred over IVIG due to rapid onset of action, but this needs to be balanced with feasibility in very young children.

## Conclusions

**Figure 1:**
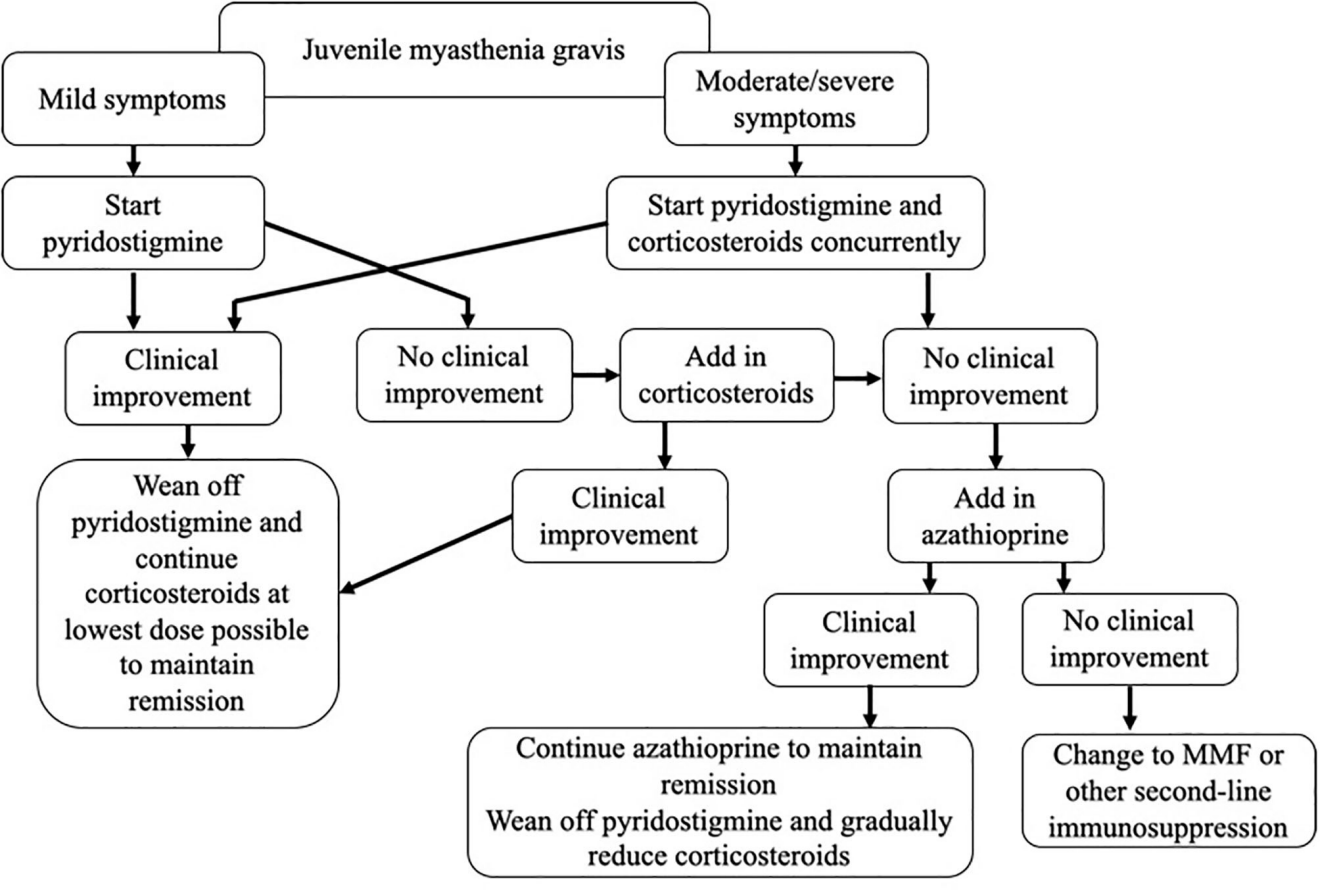
Our approach to pharmacologic therapy in juvenile myasthenia gravis.

JMG is a rare disease, and evidence-based guidelines are lacking. In this review, we have critically assessed the currently available literature, and outlined a treatment paradigm, incorporating our experience in managing these patients as a specialist referral center (Figure 1). A number of questions remain, including when to initiate both first- and second-line treatments, choosing between steroid-sparing agents, and determining the optimal dose and treatment duration. There is a need for prospective studies to properly evaluate treatment regimes, but the rarity of the disease combined with the diversity of the condition itself, and the influences of race and gender, are likely to make this challenging. In order to achieve this, collaboration across centers and the establishment of international patient registries will be needed.

### Case Studies

#### Case 1

A 2 year old Afro-Caribbean girl was referred to a pediatric ophthalmologist with unilateral resting ptosis of 30%. Her parents reported it had been present for 3 months and varied from day to day. There was evidence of fatigueability on prolonged upgaze but eye movements appeared full. She was unable to tolerate electrodiagnostic testing but AChR-Ab was positive. She was started on pyridostigmine with a good symptomatic response. She was reviewed by her ophthalmologist 6 months later and her eye exam was noted to be normal. Her parents still reported intermittent ptosis, especially when tired and at times though her left eye “drifted out.” Due to the normal exam, a decision was made to wean her pyridostigmine, however, upon discontinuation, she experienced an acute worsening of her symptoms, with new onset double vision and bilateral ptosis. At this point she was prescribed 20 mg of prednisolone daily, which was stopped at her 3 month review as she was felt to be in remission. Once off steroids, her symptoms quickly returned. She was referred to the pediatric neurology service and on exam, she was also noted to have features of generalized MG with difficulty getting up from the floor and lifting her arms above her head.

##### Comment

This case highlights a number of issues in managing JMG: the importance of not weaning treatment too early, especially when the history suggested breakthrough disease; the need for an adequate course of steroids and gradual tapering of the dose prior to discontinuation; the need for combined neurology and ophthalmology input as subtle signs of more generalized disease may have been missed at earlier assessments; and patients with ocular JMG are at greatest risk of converting to generalized disease within the first 2 years and need regular review over this time.

#### Case 2

A 13 year old Caucasian girl was referred to the neurology service with slurred speech, generalized weakness and fatigue, that had worsened over 6 weeks. She now became breathless on minimal exertion. Examination showed mild bilateral ptosis, normal eye movements, dysarthria (unable to count aloud to 10), and weakness of neck flexion and shoulder abduction. Her forced vital capacity was 50% normal. Her symptoms and exam were felt to be consistent with generalized MG and the severity raised concern for an impending myasthenic crisis. She was admitted to the neurology ward and anesthetic review was arranged. She was commenced on PLEX and concomitant pyridostigmine and oral steroids. She had a good symptomatic response to treatment and was discharged on a slow oral steroid taper, reducing by 10 mg every month to an initial maintenance of 20 mg on alternate days. Subsequent investigations showed she was AChR-Ab positive, her neurophysiology was consistent with a neuromuscular junction disorder and CT thorax was reported as showing no evidence of a thymoma.

Her first relapse occurred when her prednisolone was reduced to 30 mg alternate days, necessitating an increase in medication. She had developed a number of side-effects including weight gain and low mood. A decision was made to commence Azathioprine as a steroid-sparing agent. Her TPMT levels were normal. While her dosage was being uptitrated, her liver function became deranged, leading to discontinuation. She had another significant flare of symptoms requiring a further cycle of PLEX and her steroids were again increased. At this point she was 12 months into her diagnosis, while higher-dose steroids induced remission she was developing intolerable side-effects and was becoming depressed and withdrawn. A decision was made to refer her for thymectomy and continue intermittent PLEX prior to surgery.

##### Comment

This is a challenging case. While not treatment-refractory, our patient is steroid-dependent and was intolerant of first-line immunosuppression. We considered the addition of mycophenolate at this point, but were concerned about teratogenicity, now that she was of child-bearing age. She responded well to PLEX and given she was AChR-Ab positive with generalized disease, we felt thymectomy was the appropriate next step in management.

#### Case 3

An 8 year old Caucasian boy presented acutely with generalized weakness, shortness of breath on minimal exertion, marked dysarthria and difficulty swallowing with nasal regurgitation of fluids. His symptoms has progressed rapidly over a few weeks. Clinically his symptoms were felt to be consistent with MG. He was admitted to the pediatric ward and reviewed by the anesthetic service. He was maintained under close surveillance but a decision was made to hold off invasive ventilation. He was commenced on ChE-I, oral prednisolone and IVIG. He made good progress and was discharged home with a plan for a further course of IVIG in 4 weeks in his local hospital due the severity of his initial symptoms, and lag time for steroids to take effect. He was seen in clinic 12 months later and at this time was on maximum alternate day steroids. He was also receiving 4-weekly IVIG infusions at his local hospital. Both him and his parents reported a dramatic response to the IVIG but felt the effect wore off after about 3 weeks and his symptoms particularly fatigue became “as bad as ever.” On examination he had no weakness. Prior to his diagnosis he was said to be an outgoing boy and very involved in sports. His parents now reported he was refusing to go to school most days and no longer engaging in any extra-curricular activities. They felt the slightest thing could have him in tears. A decision was made to assess him neurologically at the time of his next infusion. While on the ward strength was noted to be normal and on further questioning he said that he kept reliving his initial hospital admission and felt that the “only reason he didn't die was because of the special protein drip.” He was felt to have evidence of post-traumatic stress disorder and appropriate psychological supports were put in place. IVIG was withheld and a gradual improvement was seen.

##### Comment

This case highlights the importance of the multidisciplinary team in managing young patients with JMG. Psychological issues need to be addressed early and the necessary supports put in place. It is not uncommon for young patients to report fatigue rather than true muscle weakness, and this is often a manifestation of an underlying mood disorder rather than their MG. Careful assessment needs to be carried out in all patients prior to using IVIG to ensure that it is being used in the appropriate setting.

## Author Contributions

KO'C was involved in the planning of the manuscript, wrote the original draft, and subsequent changes. SR was involved in the planning and review of the manuscript. JP conceived the original topic idea, and was involved in the planning and review of the manuscript. All authors contributed to the article and approved the submitted version.

## Conflict of Interest

JP is partly funded by highly specialized services to run a national congenital myasthenia service and a neuromyelitis service. She has received support for scientific meetings and honorariums for advisory work from Merck Serono, Biogen Idec, Novartis, Teva, Chugai Pharma and Bayer Schering, Alexion, Roche, Genzyme, MedImmune, EuroImmun, MedDay, Abide ARGENX, UCB, and Viela Bio and grants from Merck Serono, Novartis, Biogen Idec, Teva, Abide, MedImmune, Bayer Schering, Genzyme, Chugai, and Alexion. She has received grants from the MS society, Guthrie Jackson Foundation, NIHR, Oxford Health Services Research Committee, EDEN, MRC, GMSI, John Fell, and Myaware for research studies. The remaining authors declare that the research was conducted in the absence of any commercial or financial relationships that could be construed as a potential conflict of interest. The handling editor declared a past co-authorship with author JP.
